# Types of vegetables shape composition, diversity, and co-occurrence networks of soil bacteria and fungi in karst areas of southwest China

**DOI:** 10.1186/s12866-023-02929-3

**Published:** 2023-07-19

**Authors:** Xiaoliao Wei, Tianling Fu, Guandi He, Zhuoyan Zhong, Mingfang Yang, Fei Lou, Tengbing He

**Affiliations:** 1 College of Agriculture, Guizhou University, Guiyang, 550025 PR China; 2 Institute of New Rural Development, Engineering Key Laboratory for Pollution Control and Resource Reuse Technology of Mountain Livestock Breeding, Guizhou University, Huaxi District, Guiyang City, 550025 Guizhou Province PR China

**Keywords:** Leafy vegetable soil, Melon and fruit soil, Microorganism, Co-occurrence network, Keystone taxa

## Abstract

**Background:**

Microorganisms are of significant importance in soil. Yet their association with specific vegetable types remains poorly comprehended. This study investigates the composition of bacterial and fungal communities in soil by employing high-throughput sequencing of 16 S rRNA genes and ITS rRNA genes while considering the cultivation of diverse vegetable varieties.

**Results:**

The findings indicate that the presence of cultivated vegetables influenced the bacterial and fungal communities leading to discernible alterations when compared to uncultivated soil. In particular, the soil of leafy vegetables (such as cabbage and kale) exhibited higher bacterial α-diversity than melon and fruit vegetable (such as cucumber and tomato), while fungal α-diversity showed an inverse pattern. The prevailing bacterial phyla in both leafy vegetable and melon and fruit vegetable soils were *Proteobacteria*, *Acidobacteriota*, *Actinobacteriota*, and *Chloroflexi.* In leafy vegetable soil, dominant fungal phyla included *Ascomycota*, *Olpidiomycota*, *Mortierellomycota*, and *Basidiomycota* whereas in melon and fruit vegetable soil. *Ascomycota*, *Mortierellomycota*, *Basidiomycota*, and *Rozellomycota* held prominence. Notably, the relative abundance of Ascomycota was lower in leafy vegetable soil compared to melon and fruit vegetable soil. Moreover, leafy vegetable soil exhibited a more complex and stable co-occurrence network in comparison to melon and fruit vegetable soil.

**Conclusion:**

The findings enhance our understanding of how cultivated soil bacteria and fungi respond to human disturbance, thereby providing a valuable theoretical basis for soil health in degraded karst areas of southwest China.

**Supplementary Information:**

The online version contains supplementary material available at 10.1186/s12866-023-02929-3.

## Introduction

Karst landforms hold global significance, covering approximately 15% of continental the surface areas and accommodating around 25% of the world’s population [1, 2]. These Karst regions are characterized by substantial carbonate rock outcrops, extensive distribution limited soil volume, shallow soil layers, and slow soil formation [3]. However, karst landforms are highly vulnerable ecosystems prone to the detrimental consequences of ecological collapse and rocky desertification [4]. Ecological degradation in karst regions manifests primarily as rocky desertification, which involves the loss of soil, arable land, biodiversity compromised water resources, and deterioration of plant communities [5]. The largest karst areas in the world are primarily concentrated in southwestern China, encompassing both the southern mountains and the Yunnan-Guizhou Plateau [6]. These regions are agriculturally significant, harboring valuable soil resources [7]. Presently, southwest China faces the challenge of approximately 550,000 square kilometers of karst regions that have been subjected to severe human disturbances, including deforestation, farming, burning, and grazing [8, 9], resulting in substantial ecological deterioration associated with agricultural activities in karst areas [10]. This has led to increased agricultural practices on marginal soils, sloping lands, and ridges, accompanied by population growth and declining land productivity [11]. Consequently, the degradation of karst ecosystems, altered community functioning and accelerated land desertification have ensued, thereby impacting soil quality in the region [12]. Soil degradation precipitates a decline in soil nutrient content and microbial activity, adversely affecting soil fertility and ecological conditions [13]. Several studies indicate that rock desertification contributes to the reduction of soil nutrients, with nitrogen, phosphorus, and potassium being the primary factors driving this phenomenon [14]. Karst rock desertification represents a progressive process of land degradation that gives rise to desert-like landscapes, the depletion of endemic biomass, and a deterioration in soil quality, which diminishes with the increasing severity of rock desertification [15]. Hence, the implementation of standardized sustainable agricultural practices tailored to the specific conditions of the karst area becomes imperative [11]. Nevertheless, diverse plant species manifest unique adaptive strategies within the karst area environment [16]. Consequently, comprehending the response of soil microbes to these plant-mediated processes assumes the utmost significance for fostering sustainable agricultural development in karst area regions.

In agroecosystems, particularly within vulnerable karst ecosystems, soil microbes play pivotal roles in restoring and maintaining ecosystem health [17], as they actively contribute to essential processes and functions related to soil cycling [18]. Microbial communities are considered indicators of ecosystem stability, sustainability, and overall health. For instance, the success of revegetation endeavors heavily relies on the restoration of the microbial community [19]. Nevertheless, the composition of soil microorganisms exhibits high variability, and the intricate structure of the soil microbial community significantly influences its functioning [20]. These microorganisms thrive in symbiosis with other members of the community members, fostering beneficial interrelationships [21]. Co-occurrence networks emerged as valuable tools for investigating intricate associations among diverse microbial species [21, 22]. They have been employed as an indicator for to explore microbiome interdependencies and provide valuable insights into co-occurrence patterns and their underlying mechanisms [23]. Keystone taxa have been employed as an indicator for changes in microbial communities and compositional shifts, owing to their capacity to confer greater biological connectivity within these communities [24]. The removal of keystone taxa can have detrimental effects on microbiome stability, leading to significant alterations in microbial function and composition [25].

Existing studies primarily focus on changes in hydrological characteristics [26], plant diversity [27], and soil nutrients and quality in degraded karst ecosystems [28]. However, our understanding of how human disturbances such as the cultivation of various vegetable types, influence bacterial and fungal communities in in karst soil remains limited. Therefore, we conducted a study using 21 soil samples collected from vegetable cultivation areas in the karst region, encompassing uncultivated soil as well as soils where cabbage, kale, cucumber, and tomato were grown. The main objective of this research was to assess the impact of planting different vegetable types on the soil microbial community in the karst area. We evaluated microbial diversity, composition, co-occurrence networks, and environmental factors in karst area to investigate these effects.

## Materials and methods

### Study area and soil sampling

The study area is located in Shiliping, Jingnan Town, Xingyi City, Guizhou Province, China (24°58′0″N-25°1′0″N, 104°50′0″E-104°55′0″E) is the location of the research area. It exhibits typical karst landforms and a humid subtropical monsoon climate. The cultivated land spans approximately around 2 km^2^ (Figure S1) and has been under intensive management since 2013 until the time of sampling. The main vegetable types grown on the cultivated land include kale (*Brassica oleracea var. capitata L.*), cabbage (*Brassica rapa var. glabra Regel*), tomato (*Lycopersicon esculentum Miller*), and cucumber (*Cucumis sativus L.*). Detailed management information can be found in Table S1 of the supplementary file. In this study, we classified the soils of cabbage and kale as leafy vegetable soil, while the soils of cucumber and tomato were categorized as melon and fruit soil. Soil samples were collected in December 2018 from 21 sites representing different vegetable types: cabbage (harvesting stage, n = 6), kale (harvest phase, n = 6), cucumber (harvest phase, n = 3), tomato (harvest phase, n = 3), and uncultivated soil (serving as a control treatment; dominated by withering-phase tiny herbs, n = 3). A total of 21 bulk soil samples were collected, comprising leafy vegetables (cabbage and kale) and melon and fruit vegetables (cucumber and tomato). The sampling method and preservation conditions were consistent with our previous study [29]. Some of the collected samples were used for microbiological analysis, while the remaining portion was utilized for determining soil environmental factors.

### Measurement of soil environmental factors

The pH, organic matter (OM), alkaline hydrolyzable nitrogen (AN), available phosphorus (AP), available kalium (AK), total nitrogen (TN), total phosphorus (TP), and total kalium (TK) of soil were determined by the potentiometric method, potassium dichromate-sulfuric acid heating method, alkaline hydrolysis diffusion method, ammonium acetate leaching-atomic absorption method, molybdenum antimony anti-colorimetric method, kjeldahl method, nitric acid-perchloric acid-hydrofluoric acid digestion method, and nitric acid-perchloric acid-hydrofluoric acid digestion method, respectively [30]. The urease (UR), catalase (CA), sucrase (SU), and phosphatase (PHO) of soil were determined by Sodium phenolate colorimetric method, potassium permanganate titration, 3,5-dinitrosalicylic acid colorimetric method, disodium phenyl phosphate colorimetric method, respectively [31, 32]. “List of abbreviations” can be found in the Supplementary files (word.doc).

### Extraction of soil dna and sequencing of amplicon

Microbial genomic DNA from all soil samples was extracted using the soil-specific FastDNA® Spin Kit, following the manufacturer’s instructions [33]. The purity and quantity of the DNA were assessed using a NanoDrop2000 spectrophotometer and 1.0% agarose gel electrophoresis. For amplification, theV3-V4 region of the bacterial 16 S rRNA gene was targeted using 338 F and 806R primers while ITS1 region of the fungal ITS rRNA gene was amplified using the ITS1F and ITS2R primers. Three replicates of each sample were subjected to PCR amplification using specific PCR programs outlined in Table S2. The resulting PCR products were then pooled in equimolar ratios and sequenced using an Illumina MiSeq platform. The raw sequencing data have been deposited in the NCBI Sequence Read Archive database under the accession number PRJNA865810.

### Data collection and statistical analysis

We employed QIIME [34] to demultiplex and quality-filter the raw fastq files, with the aid Details of the procedures can be found in Table S2. Operational taxonomic units (OTUs) for bacteria and fungi were generated using UPARSE with a 97% sequence similarity threshold [35]. Chimeric sequences were identified and removed using UCHIME [35, 36]. After filtering out OTUs that did not belong to the soil bacterial and fungal communities, we obtained a total of 8,475 bacterial and 2,812 fungal OTUs. Taxonomic classification of the representative sequences of OTUs at a 97% similarity level was performed using the RDP classifier Bayesian method. Bacterial and fungal taxonomic analyses were conducted using the SILVA and UNITE databases, respectively. To visualize the data, we utilized various R packages, including pheatmap, ggplot2, ggvenn, reshape2, and ggplot2. These packages were employed to generate clustered heatmaps, boxplots, Venn diagrams, stacked percentage plots, and relevance heat maps. Correlation analysis and ANOVA (analysis of variance) with Duncan’s multiple range tests (P < 0.05) were conducted using SPSS statistical software (Version 25.0). For network analysis representative OTUs present in all soil samples with a mean relative abundance of 0.1% in each group were selected based on Spearman (Spearman’s r <- 0.7 or r > 0.7; P < 0.05). The igraph package in R was used to build The co-occurrence network was constructed using the igraph package in R, and Gephi software was used for visualization [37]. Keystone taxa were identified based on the descriptions provided by previous studies [38, 39].

## Results

### Effect of planting different vegetable types on environmental factors

**Fig. 1:**
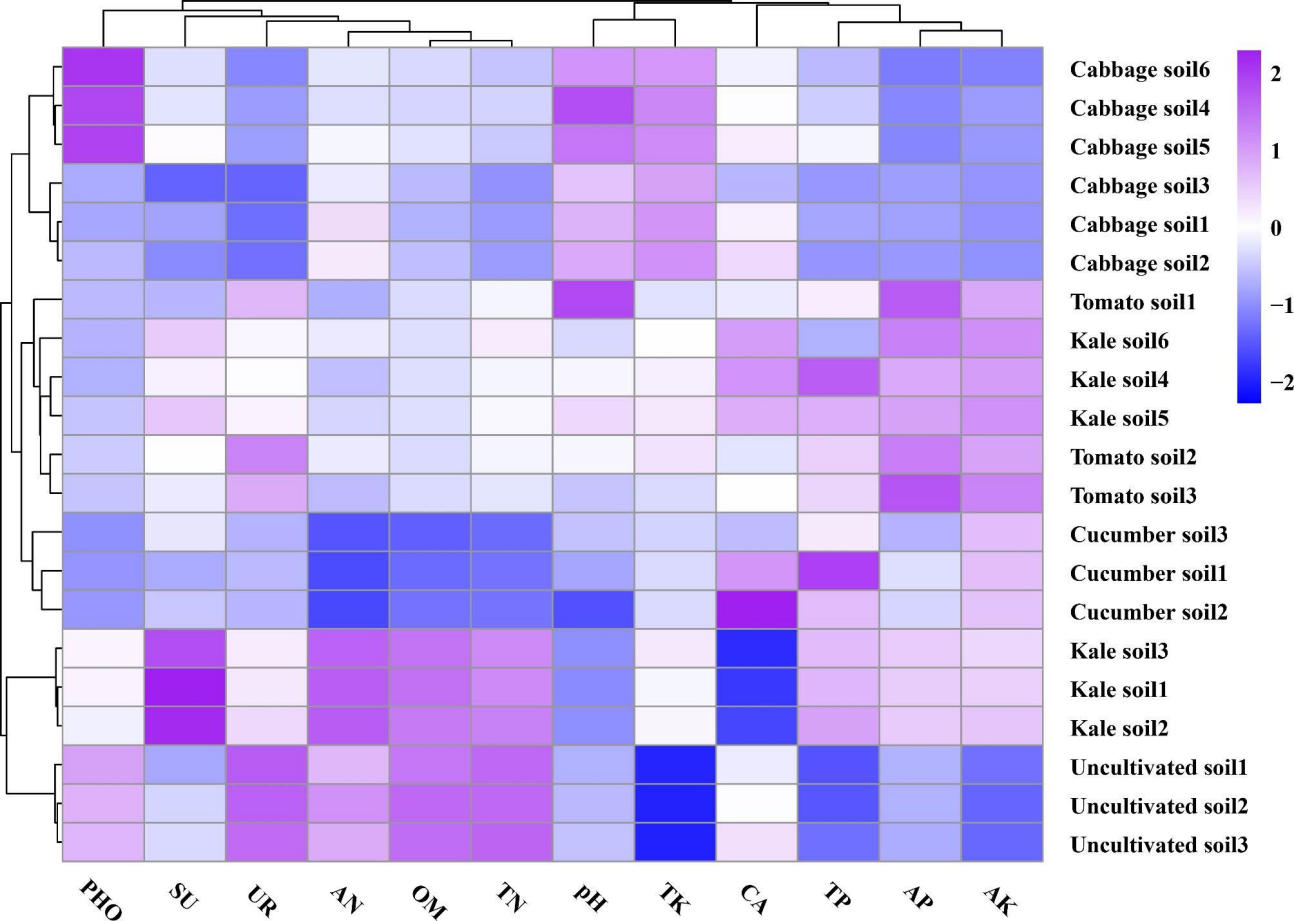
Effects of planting different vegetable types on environmental factors. Notes: pH represents hydrogen ion concentration, OM represents organic matter, AN represents alkaliehydrolyzable nitrogen, AP represents available phosphorus, AK represents available kalium, TN represents total nitrogen, TP represents total phosphorus, TK represents total kalium, CA represents catalase, UR represents urease, SU represents sucrase, and PHO represents phosphatase

The soil parameters showed significant variations among different types of vegetable planting (Fig. 1 and Table S3). Notably, pH exhibited significant differences across the vegetable soils, with the lowest value (7.03) observed in cucumber soil and the highest value (7.62) in cabbage soil (Table S3). The concentrations of AN, TN, and OM were reduced in soils planted with cucumber, tomato, kale, and cabbage compared to uncultivated soil. Among these indicators cucumber soil had the lowest concentrations of AN, TN, and OM, while kale soil had the highest concentrations (Table S3). On the other hand, the planting of cabbage, kale, cucumber, tomato increased the contents of AK, TK, AP, and TP in the soils. Specifically, cabbage and kale soils exhibited higher TK concentrations compared to cucumber and tomato soils. TK concentrations displayed significant variations among the different vegetable types (Table S3). The highest TK concentration (12.09 g·kg^− 1^) was found in cucumber soil, while the lowest TK concentration (8.33 g·kg^− 1^) was observed cabbage soil (Table S3).

The concentrations of AK and TP in the cabbage soils were significantly lower than those in the tomato and cucumber soils, and the concentrations of AK and TP in the tomato and cucumber soils were not significantly different from those in the kale soil. The concentration of AP was the highest (147.09 mg·kg^− 1^) and lowest (19.47 mg·kg^− 1^) in the tomato and cabbage soils, respectively. However, there was no obvious regularity in the variation of the concentration of AP (Table S3).

The activity of CA did not show significant variation among different types of vegetable planting. However, UR, SU, and PHO activities exhibited significant variations across the different soils; The highest UR activity (2.72 NH_3_-N, mg·g^− 1^·(24 h)^−1^) was found in was observed in tomato soil, while the lowest UR activity (0.68 NH_3_-N, mg·g^− 1^·(24 h)^−1^) was found cabbage soil, The highest SU activity (38.30 Glucose, mg·g^− 1^·(24 h)^−1^) was observed in kale soil, while the lowest SU activity (20.37 Glucose, mg·g^− 1^·(24 h)^−1^) was found cabbage soil (Table S3). Moreover, the highest PHO activity (0.64 Phenol, mg·g^− 1^·(24 h)^−1^) was observed in cabbage soil, while the lowest PHO activity (0.11 Phenol, mg·g^− 1^·(24 h)^−1^) was found cucumber soil (Table S3), respectively. Overall, the cultivation of cabbage, kale, cucumber and tomato resulted in changes in soil environmental factors, with lower concentrations of most indicators observed in the cucumber and tomato soils compared to cabbage and kale soils. Additionally, the cluster heatmap analysis revealed that cucumber and tomato soils clustered together, indicating their relatively similar environmental characteristics (Fig. 1).

### Effect of planting different vegetable types on bacterial and fungal otus and alpha diversity

**Fig. 2:**
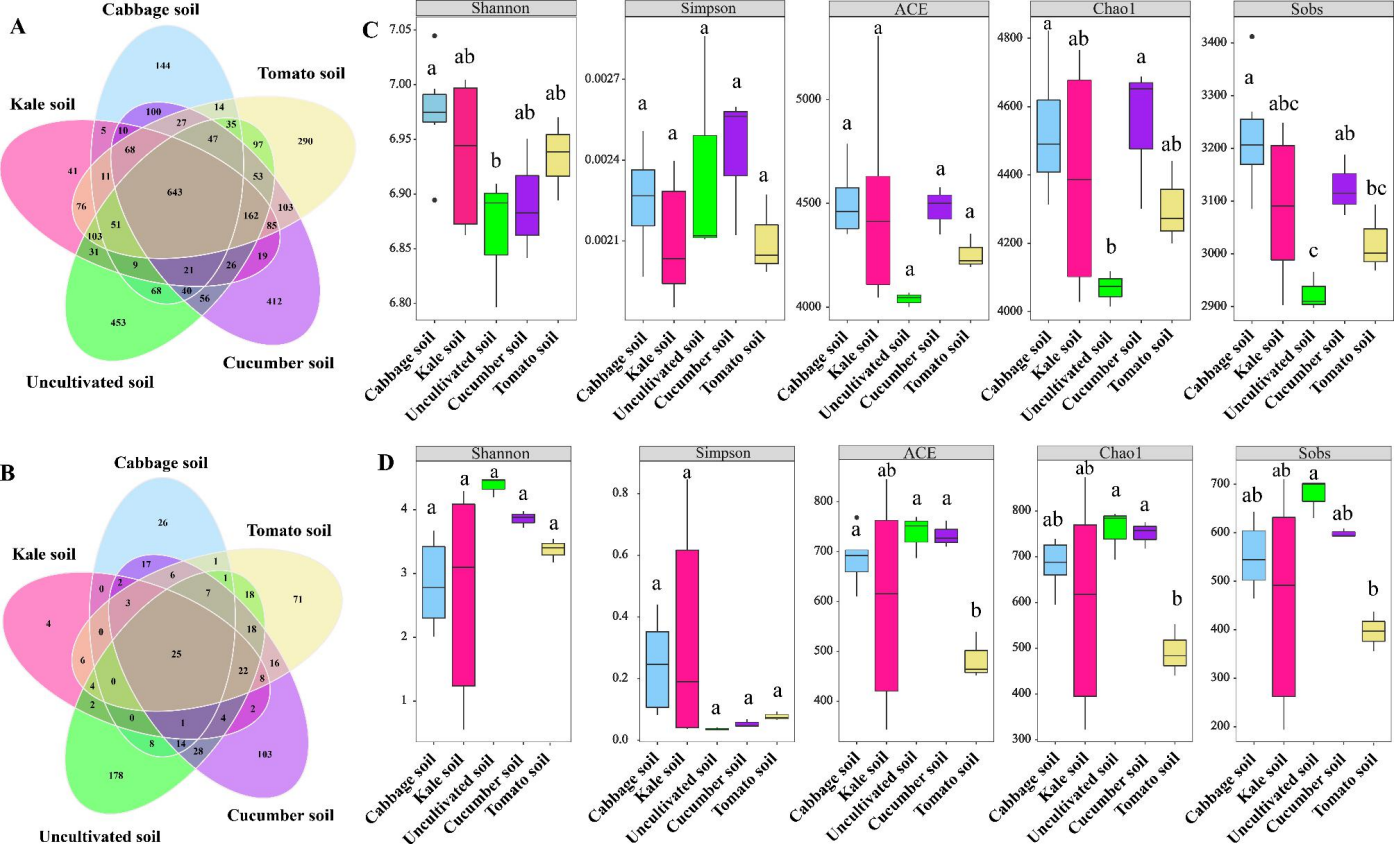
The impacts of planting different vegetable types on bacterial and fungal operational taxonomic units (OTUs) and alpha diversity. **A** and **B** represent the Venn analysis of bacterial and fungal OTUs, respectively. **C** and **D** represent the Analysis of Variance (ANOVA) of bacterial and fungal alpha diversity, respectively. Notes: ANOVA refers to Analysis of Variance. The different lowercase letters above the box plots indicate significant differences between different soil groups based on one-way ANOVA with Duncan’s multiple range tests (P < 0.05)

The distribution of soil bacterial and fungal operational taxonomic units (OTUs) varied among the different vegetable planting types with bacterial OTUs ranging from 1,293 to 1,895 (Fig. 2A) and fungal OTUs ranging from 83 to 330 (Fig. 2B). Bacteria exhibited a higher number of OTUs compared to fungi. While there were differences in the number of OTUs among soils with cabbage, kale, uncultivated, cucumber, and tomato, there were more common bacterial OTUs across all five groups than unique bacterial OTUs specific to each group (Fig. 2A). In contrast, fungal OTUs displayed an opposite trend (Fig. 2B). This suggests that the bacterial community composition was relatively similar among soils with cabbage, kale, uncultivated, cucumber, and tomato, while the, while the fungal community composition showed some differences (Fig. 2A, B).

Compared to uncultivated soil, the planting of cabbage, kale, cucumber, and tomato increased the Shannon, ACE, Chao1, and Sobs indices of soil bacterial α-diversity. Significant variations in the Shannon, Chao1, and Sobs indices were observed among different vegetable types planting except for the Shannon index of kale, cucumber, and tomato, no significant difference in Chao1 index of kale and tomato, and no significant difference in the Sobs index of kale and tomato; However, the Simpson and ACE indices of bacterial diversity did not significantly differ among soils different with different vegetable types (Fig. 2C and Table S4). These findings indicate that plant cultivation can influence soil bacterial alpha diversity. The Shannon and Sobs indices of soil bacterial α-diversity were the lowest in uncultivated soil and highest cabbage soil, while the Chao1 index of bacterial α-diversity was lowest in uncultivated soil and highest cucumber soil (Fig. 2C and Table S4). The Shannon and ACE indices of bacterial α-diversity in the cabbage and kale soils were greater than those in the cucumber and tomato soils, and the Sobs index of bacterial α-diversity in the cabbage soil was greater than that in the cucumber and tomato soils, showing plant cultivation did not significant effect on fungal α-diversity. Compared with uncultivated soil, the planting of cabbage, kale, cucumber, tomato decreased the Shannon, ACE, Chao1, and Sobs indices of soil fungal α-diversity; the planting of cabbage, kale, and cucumber, and tomato increased the Simpson index of soil fungal α-diversity, and these indicators did not significantly different in soils with different vegetable types planting except for Sobs index of tomato, ACE index of tomato, and Chao1 index of tomato (Fig. 2D and Table S4) Moreover, the Chao1, ACE, and Sobs indices of fungal α-diversity in tomato soil were lower than those in cabbage and kale soils (Fig. 2D and Table S4). In summary, leafy vegetable soil exhibited higher bacterial α-diversity compared to melon and fruit vegetable soil, while the opposite trend was observed for fungal α-diversity.

### Analysis of bacterial and fungal community composition

**Fig. 3:**
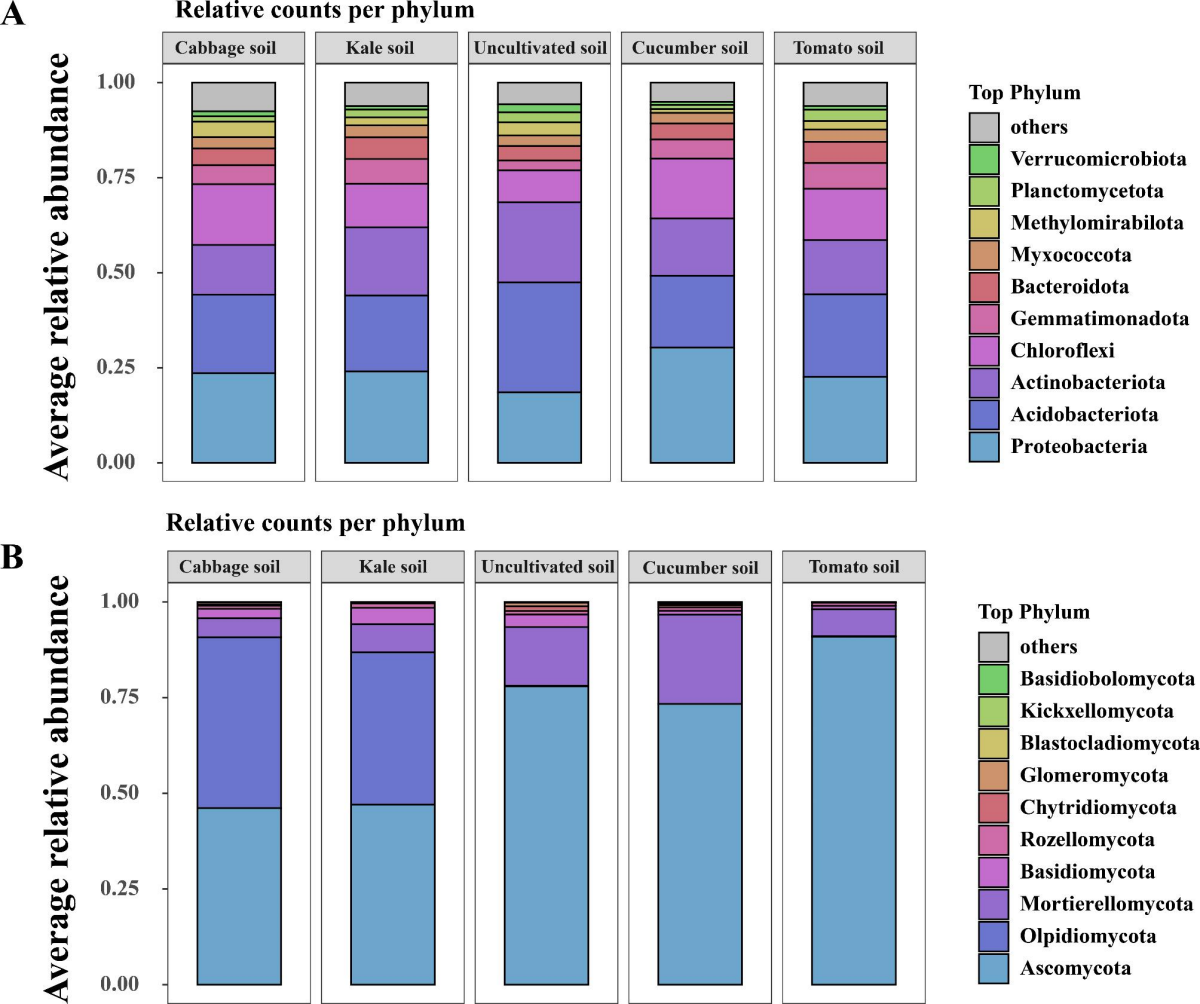
Composition and beta diversity of bacterial and fungal communities. Notes: **A** and **B** represent the composition of soil bacterial and fungal communities at the phylum level, respectively. **C** and **D** represent the beta diversity of soil bacterial and fungal communities based on operational taxonomic units (OTUs).

A total of 45 bacterial phyla and 13 fungal phyla were identified in the study (Fig. 3 and Table S5). The dominant bacterial phyla in soils with different vegetable planting types were *Proteobacteria, Acidobacteriota, Actinobacteriota, and Chloroflexi* which collectively accounted for over 70% of the bacterial phyla. Other major bacterial phyla included *Gemmatimonadota, Bacteroidota, Myxococcota, Methylomirabilota, Planctomycetota*, and *Verrucomicrobiota* (Fig. 3A and Table S5). Among these, the first four abundant phyla of bacteria in the cabbage, kale, cucumber, and tomato soils, *Proteobacteria* exhibited significant variations across different plant types with the lowest relative abundance (18.55%) in uncultivated soil and the highest (30.29%) in cucumber soil (Fig. 3A and Table S5). In terms of fungal phyla, the dominant one in cabbage and kale soils were *Ascomycota, Olpidiomycota, Mortierellomycota*, and *Basidiomycota* (Fig. 3B and Table S5). In cucumber and tomato soils, the dominant fungal phyla were *Ascomycota, Mortierellomycota, Basidiomycota* and *Rozellomycota*. In uncultivated soil, the dominant fungal phyla were *Ascomycota, Mortierellomycota*, *Basidiomycota*, and *Chytridiomycota*. These four dominant fungal phyla accounted for > 97% of the phyla in soils with different vegetable planting types. Other major fungal phyla were *Rozellomycota*, *Chytridiomycota*, *Glomeromycota*, *Blastocladiomycota*, *Kickxellomycota*, and *Basidiobolomycota* (Fig. 3B and Table S5). Interestingly, the relative abundance of *Ascomycota* was lower in leafy vegetable soils (cabbage: 46.17%, kale: 47.08%) compared to melon and fruit vegetable soils (cucumber: 73.33%, tomato: 90.91%), Furthermore, the relative abundance of *Ascomycota* in tomato soil (90.91%) was significantly higher than that in cabbage (46.17%) and kale (47.08%) soils (Fig. 3B and Table S5).

### Bacteria had a wider distribution than fungi

**Fig. 4:**
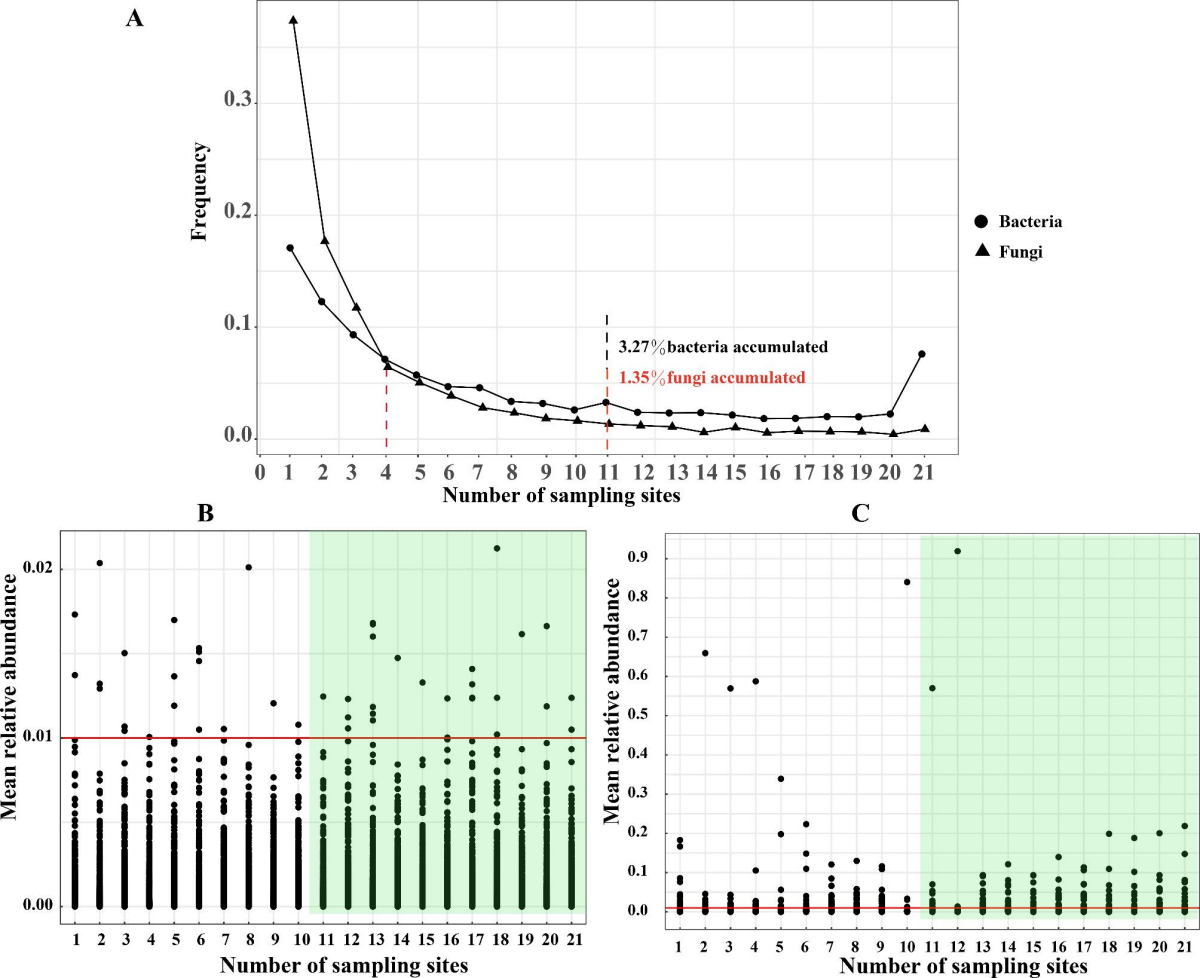
The distribution of operational taxonomic units (OTUs) of soil bacteria and fungi in karst areas. **A** represents the frequency distribution of bacterial and fungal OTUs. **B** and **C** represent OTUs with a relative abundance exceeding 1% for bacteria and fungi, respectively. Notes: The green highlighted area indicates the distribution of OTUs with a relative abundance higher than 1% (observed at sampling sites with a count of 11 or more)

We analyzed the distribution of bacterial and fungal communities in the soils by counting the number of sample points for each OTU (Fig. 4A). The results showed that the majority of fungi were found in sample points with OTU counts of ≤ 4 (over 70%), while bacteria exhibited higher frequencies than fungi in sample points with OTU counts > 4 (Fig. 4A and Table S6). Notably, a higher proportion of bacterial OTUs (3.27%) were distributed in more than half of the sampling sites (11 sites) compared to fungi (1.35% of all fungal OTUs), indicating that fungi generally had more restricted distribution patterns than bacteria (Fig. 4A). Interestingly, at 19 sampling points, the frequency distributions of bacteria and fungi were 1.98% (of all bacterial OTUs) and 0.64% (of all fungal OTUs), respectively. However, when the number of sampling points increased to 21, the frequency distributions of bacteria and fungi were 7.60% (of all bacterial OTUs) and 0.89% (of all fungal OTUs), respectively (Table S6). This suggests that as the number of sampling points increased, bacteria exhibited a wider distribution range compared to fungi. Furthermore, when the number of sampling points was greater than half (≥ 11), fungi had a higher number of OTUs with a relative abundance of > 1% (6.01% for the fungi vs. 0.31% for bacteria) (Fig. 4B, C). We also identified core OTUs based on our criteria: OTUs present in each group of samples with a mean relative abundance > 0.1%. The ratio of core OTUs to all OTUs was 5.11% for bacteria and 3.59% for fungi, further supporting the wider distribution of bacteria compared to fungi. Additionally, the core OTUs were commonly found across multiple phyla, including *Proteobacteria*, *Chloroflexi*, *Acidobacteriota*, and *Actinobacteriota* for bacteria and *Ascomycota*, *Basidiomycota*, and *Mortierellomycota* for fungi (Table S7).

### Correlation analysis of bacterial and fungal communities with environmental factors

**Fig. 5:**
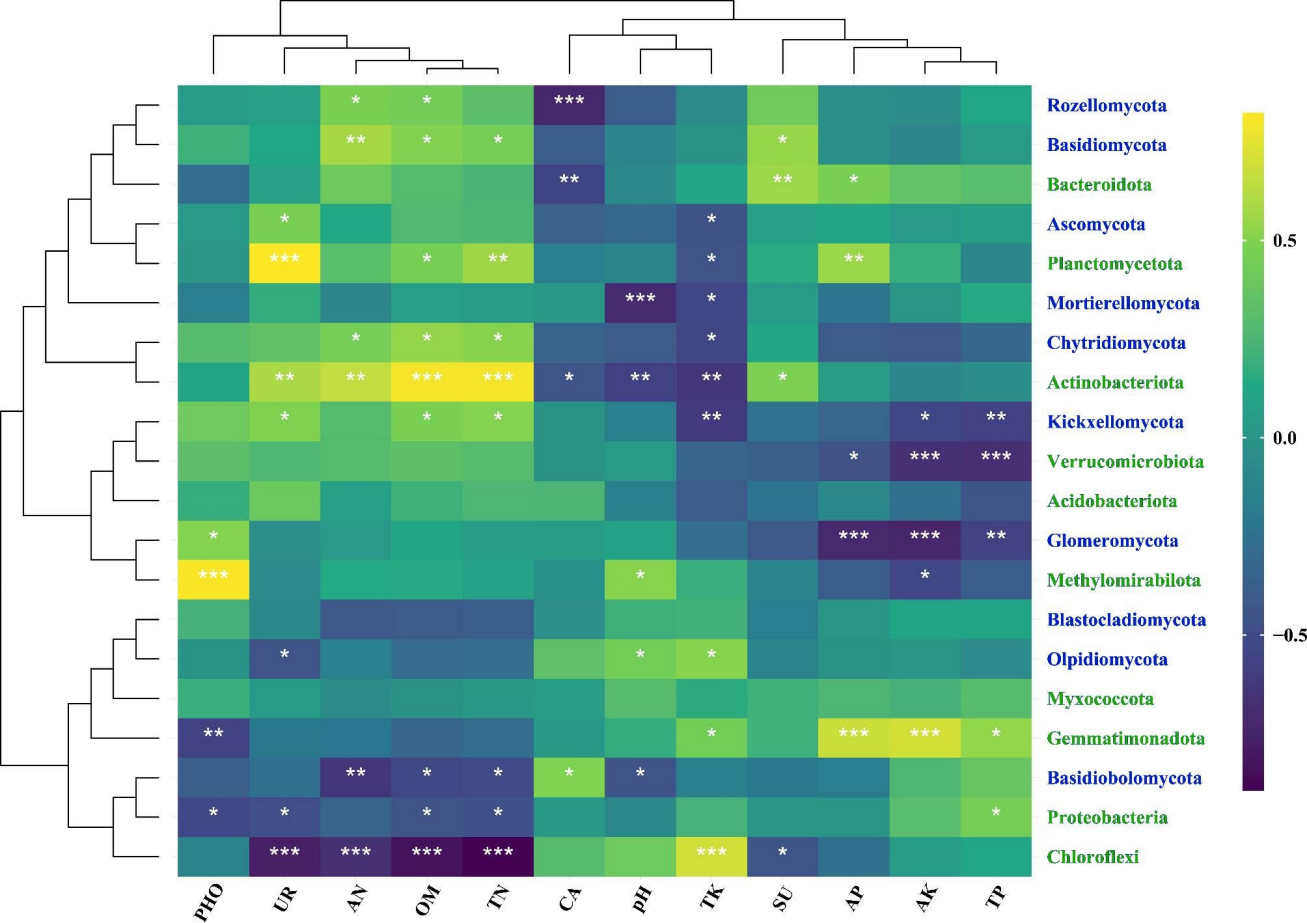
Pearson correlation analysis of bacterial and fungal communities with environmental factors. Notes: “***” shows significance at p ≤ 0.001, “**” at 0.001 < p ≤ 0.01, “*” at 0.01 < p ≤ 0.05. The green and blue fonts represent the top 10 dominant bacterial and fungal phyla based on relative abundance, respectively

In order to examine the correlationship between soil environmental factors and microbial communities, we conducted a correlation analysis between the top 10 bacterial and fungal phyla in terms of relative abundance and the environmental factors. The results revealed significant correlations between environmental factors and the bacterial and fungal communities. The bacterial community showed stronger positive correlations with environmental factors compared to the fungal communities, particularly with *Actinobacteriota*, *Planctomycetota*, and *Gemmatimonadota* (Fig. 5). *Planctomycetota* exhibited significant positive correlations with UR, OM, TN, and AP while showing a significant positive correlation with TK. Strong positive association were observed between *Bacteroidota* and SU and AP, whereas a substantial negative correlation was found with CA., Actinobacteriota showed significant positive associations with SU, UR, AN, OM, and TN, but significant negative correlations with pH, CA, and TK. *Proteobacteria* exhibited a significant positive with TP but a significant negative correlation with PHO, UR, OM, and TN. *Chloroflexi* demonstrated a significant negative correlation with UR, OM, TN, and SU, but a significant positive correlation with TK (Fig. 5 and Table S8). Notably, *Actinobacteriota* showed significant positive relationships with UR, AN, OM, TN, and SU showed significant positive relationship, while showing a significant negative relationship with *Chloroflexi*. Only a few fungal communities showed a significant positive correlation with environmental factors, such as *Basidiomycota*, *Chytridiomycota*, and *Kickxellomycota*. *Basidiomycota* exhibited significant positive correlations with AN, OM, TN, and SU. *Chytridiomycota* showed significant positive correlations with AN, OM, and TN, while *Kickxellomycota* indicated significant positive correlations with UR, OM, and TN (Fig. 5 and Table S8). On the other hand, *Glomeromycota*, *Kickxellomycota*, and *Mortierellomycota* displayed significant negative correlations with environmental factor. *Glomeromycota* showed significant negative correlations with AP, AK, and TP, while *Kickxellomycota* displayed significant negative correlations with TK, AK, and TP. *Mortierellomycota* exhibited significant negative correlations with pH, and TK (Fig. 5 and Table S8). Overall, our findings indicate that the bacterial and fungal communities mainly primarily showed significant positive correlations with UR, AN, OM, TN, and SU, while displaying significant negative correlations with CA, pH, TK, and AK. Additionally, we observed significant positive correlations between. soil physicochemical properties (AN, OM, and TN), soil enzyme activities (UR and SU), and the majority of the studied factors. Furthermore, the content of AN and TN showed a significant positive correlation with the content of OM (Table S9).

### Analysis of co-occurrence network

To investigate the impact of planting of leafy vegetables and melon and fruit vegetables on bacterial-fungal co-occurrence networks, we analyzed the interkingdom co-occurrence network of bacteria and fungi, as well as the intra-kingdom co-occurrence networks of fungi-fungi and bacteria-bacteria.

**Fig. 6:**
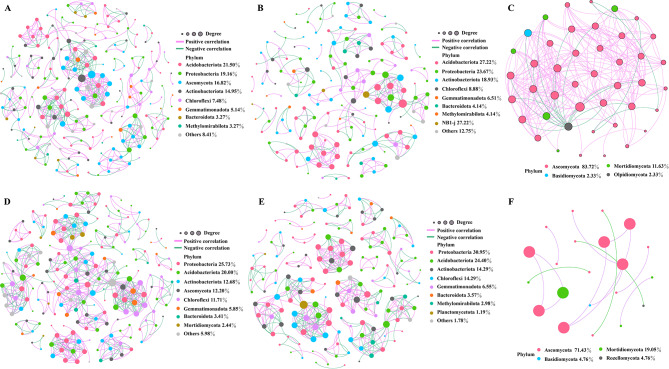
Analysis of co-occurrence networks in leafy vegetable and melon and fruit vegetable soils. **A** represents the bacterial-fungal interkingdom co-occurrence network of leafy vegetable soil. **B** and **C** represent the intra-kingdom co-occurrence networks of bacteria-bacteria and fungi-fungi, respectively, in leafy vegetable soil. **D** represents the bacterial-fungal interkingdom co-occurrence network of melon and fruit vegetable soil. **E** and **F** represent the intra-kingdom co-occurrence networks of bacteria-bacteria and fungi-fungi, respectively, in melon and fruit vegetable soil. Notes: The nodes in the network are color-coded based on the phyla of the microbes. Edges represent correlations between nodes, with positive correlations shown in purple and negative correlations shown in green

Firstly, we examined the bacterial-fungal network. In leafy vegetable soil, the bacterial-fungal network consisted of 214 nodes (80.37% for bacteria and 19.63% for fungi) and 503 edges (59.05% for bacteria-bacteria, 27.24% for bacteria-fungi, and 13.72% for fungi-fungi) (Fig. 6A). Of these edges, 58.65% showed positive correlations. In the melon and fruit vegetable soil, the bacterial-fungal network had 205 nodes (83.90% for bacteria and 16.10% for fungi) and 596 edges (75.34% for bacteria-bacteria, 21.32% for bacteria-fungi, and 2.35% for fungi-fungi) (Fig. 6B). with 57.21% of the edges were positively correlated showing positive correlations. We identified 61 OTUs (including 56 bacterial and fungal OTUs) as keystone OTUs in the leafy vegetable soil network (Figure S2A and Table S10), and 42 OTUs (including 35 bacterial and 7 fungal OTUs) in the melon and fruit vegetable soil network (Figure S2B and Table S11). The leafy vegetable soil network exhibited greater complexity and stability compared to the melon and fruit vegetable soil network the melon and fruit vegetable soil network, as evidenced by the number of nodes, percentage of bacteria-fungi edges, and presence of keystone OTUs.

Next, we analyzed the bacterial-bacterial and fungal-fungal networks. The nodes and edges of the bacterial-bacterial network were 169 and 297, respectively, in the leafy vegetable soil, the nodes and edges of fungal- fungal network were 43 and 25 respectively, in the leafy vegetable soil (Fig. 6B, C). 66 OTUs of the bacterial-bacterial network were identified as keystone taxa (Figure S2C and Table S12), whereas only 5 OTUs were predicted as keystone taxa in the fungal-fungal network (Figure S2D and Table S12). The nodes and edges of the bacterial-bacterial networks of the melon and fruit vegetable soil were 168 and 449, respectively, and the nodes and edges of the fungal-fungal networks of the melon and fruit vegetable soil were 21 and 14, respectively (Fig. 6E, F). 37 OTUs of the bacterial-bacterial network were identified as keystone taxa (Figure S2E and Table S13), whereas 21 OTUs of the fungal-fungal network were identified as keystone taxa (Figure S2F and Table S13). This indicates that the bacterial-bacterial network has greater complexity and stability than the fungal-fungal network in terms of nodes, edges, and keystone taxa of the network.

## Discussion

Soil microbiological activities have impact on soil fertility and plant growth as they enhance the activity of enzymes, hormones, and nutrient cycling essential for optimal plant growth and development [40]. Our research findings indicate a strong correlation between soil environmental factors and plant type. In comparison to uncultivated soil, the cultivation of cabbage, kale, cucumber, and tomato resulted in reduced soil concentrations of OM, AN, TN, UR, SU, and PHO (Fig. 1 and Table S1). Several reasons can explain these observations. Firstly, most of the above-ground biomass of cabbage, kale, cucumber, and tomato biomass (above ground) is removed by farmers after harvest, while the plant residues in uncultivated soil decompose, leading to an increase in organic matter content [41]. Consequently, nitrogen and carbon accumulate in the soil, and higher carbon content of carbon promotes nitrogen content accumulation [42]. Additionally, we observed a strong positive connection relationship between AN, TN, and OM content (Table S11), which is consistent with findings from different grain soils in a karst region [43].

Secondly, compared to uncultivated soil, both the leafy vegetable soil and the melon and fruit vegetable soil are subjected to intensive cropping patterns, resulting in low nutrient use efficiency and soil degradation, as previous studies have reported [44, 45].

Thirdly, the presence of plastic mulch residues and disinfectants negatively affects the soil. Plastic mulch residues degrade into various-sized particles, including macro, micro, and nano particles, altering soil structure transport, and reducing soil permeability [46–48]. thereby influencing the microbiological, physical, and chemical characteristics of the soil [49]. Microplastics have also been shown to significantly decrease sucrase activity in the soil [50]. The environment of the soil may be negatively affected by soil disinfectants and their breakdown products [51]. Iprodione has the potential to hinder the activity of enzymes participating in the carbon, nitrogen, sulfur, and phosphorus cycles when it is consistently utilized [9]. Conversely, the concentrations of AP, TP, AK, and TK in the soils of cabbage, kale, cucumber, and tomato plants frequently exceeded those found in the uncultivated soil. The higher levels can be attributed to the following factors: ① The soils of cabbage, kale, cucumber, and tomato crops were primarily treated with organic fertilizer, specifically fresh pig manure that underwent fermentation, resulting in a rich in phosphorus content[52]. ② Additionally, these soils received compound fertilizer applications that contained significant amounts of potassium. Furthermore, and cucumber and tomato soils were also supplemented with potassium sulfate. Overall, the melon and fruit vegetable soil (cabbage and kale soils) exhibited lower contents of TN, TK, OM, AN, SU, and PHO compared to the leafy vegetable soil (cucumber and tomato soils) (Table S3), indicating that leafy vegetable soil is more nutrient-rich than melon and fruit vegetable soil. This can be attributed to three factors. Firstly, the amount of disinfectants in melon and fruit vegetable soil is higher compared to the leafy vegetable soil. Previous research has demonstrated the detrimental effects of soil disinfectants and their degradation byproducts on the soil ecosystem [9, 51], and more soil disinfectants mean more degradation metabolites. ② The use of pesticides in the melon and fruit vegetable soils exceeds that in the leafy vegetable soils. Research has indicated that the application of pesticides can adverse effects on soil, and the magnitude of the effects depends on the dosage of pesticides applied [53]. ③ In comparison to leafy vegetable, melon and fruit vegetables are more prone to soil-borne diseases, which can disrupt the functioning of certain microorganisms, subsequently, impact nutrient cycling in the soil [54, 55].

Plant disease control and soil structure management heavily rely on microbial populations [41]. A diverse microbial community contributes to creating favorable growing conditions and enhancing crop productivity [56]. Consistent with previous studies [57], our research identified *Proteobacteria*, *Acidobacteriota*, *Actinobacteriota*, and *Chloroflexi* as the predominant bacterial phyla in the soils of cabbage, kale, cucumber, and tomato (Fig. 2A and Table S5). In the soils of cabbage and kale soils, the dominant fungal phyla were *Ascomycota*, *Olpidiomycota*, *Mortierellomycota*, and *Basidiomycota*, whereas cucumber and tomato soils were dominated by *Ascomycota*, *Mortierellomycota*, *Basidiomycota*, and *Rozellomycota* (Fig. 2B and Table S4). The variation dominant fungal phyla can be attributed to several factors. ① Frequent soil disturbances increase litter litter quantity, root exudates, organic matter input, and lowered the soil C:N ratio compared to uncultivated soil; thus, promoting the development of community-specific roles for the topsoil microbial population [58]. ② Plant residues introduced through cultivation influence the decomposition process, with fungi playing a crucial role in decomposing plant and animal residues [59]. However, the composition and quantity of phytodetritus differ depending on the vegetation type, leading to variations in the types of fungi involved in phytodetritus decomposition; thus, influencing dominant fungal phyla to some extent. ③ Root exudates have an impact on the microbial community with different plant species affecting soil organic matter through the type and quantity of root exudation and litter breakdown [60]. Fungal taxa play a key role in regulating soil organic matter turnover, and fluctuations in organic matter content can lead to changes in the dominant fungal phyla [59, 61]. ④ The similarity in dominant fungal phyla between cabbage and kale soils and cucumber and tomato soils could be influenced by agricultural management practices [62]. In our study, cabbage and kale soils shared similar agricultural management practices, while cucumber and tomato soils exhibited similar agricultural management practices, potentially explaining this the phenomenon. It is also possible that different crops exhibit specific responses to the karst landscape [5]. Furthermore, we observed that the relative abundance of Ascomycota was lower in the leafy vegetable soils (cucumber:73.33%, tomato: 90.91%) compared to melon and fruit vegetable soil (cabbage: 46.17%, kale: 47.08%) (Fig. 3B and Table S5). Previous studies have linked higher relative abundance of Ascomycota to several soil-borne diseases, with significantly higher levels observed in diseased soils compared to healthy soils [63]. Cucumbers and tomatoes are generally more susceptible to soil-borne diseases, such as root rot and fusarium wilt [64]. Additionally, we found that soil bacteria exhibit a broader ecological niche distribution compared to fungi, potentially giving bacteria an advantage in competing for terrestrial unstable carbon resources [65]. The distribution patterns of soil microorganisms vary, with bacteria showing a wider distribution compared to archaea under the influence of salinity in the Bohai Sea area [36]. In our study of the karst region, bacteria were also found to have a broader distribution than fungi in soil samples (Fig. 4), suggesting a general trend of bacteria being more widely distributed in soils compared to fungi and archaea.

Leafy vegetable soil exhibits higher bacterial α-diversity compared to melon and fruit vegetable soil, while fungal α-diversity showed the opposite trend. However, there was no significant difference observed in the fungal alpha diversity indices (Fig. 2C, D and Table S4). Higher microbial diversity is indicative of a more stable ecological environment [66], suggesting that leafy vegetable soil possesses a more stable ecosystems than melon and fruit vegetable soil. It has been demonstrated that the pattern of bacterial cross-habitat distribution patterns of bacteria are more strongly influenced by habitat type compared to fungi in the environment [65]. Furthermore, our correlation analysis revealed that bacteria show stronger associations with soil environmental parameters than fungi (Fig. 5 and Table S9), which aligns with the findings of our previous study [29]. Complex networks, as described by Santolini and Barabási [67], are more resilient to external disturbances. Keystone taxa refer to intricately interconnected groups of taxa that play vital roles in the microbial ecosystem. The absence of keystone taxa can negatively impact the microbial community, even when their presence does not directly influence the structure or function of the microbial community [25, 68]. Our study findings indicate that the network of leafy vegetable soil exhibits greater complexity and stability compared to the network of melon and fruit vegetable soil network (Fig. 6 and Figure S2). Overall, our results suggest that the cultivation of leafy vegetables is more advantageous than that of melon and fruit vegetables in promoting the establishment of robust soil microbial communities, as evidenced by the analysis of soil environmental factors, microbial diversity, and microbial co-occurrence networks. Furthermore, it is important to note that we did not investigate the functional aspects of soil microorganisms in this study. Future research endeavors will incorporate macrogenomic and other methodologies to investigate the functional roles of soil microorganisms in both leafy vegetable and melon and fruit vegetable soils.

## Conclusion

In conclusion, our research revealed that the cultivation of cabbage, kale, cucumber, and tomato had a significant impact on both microbial communities and environmental factors in the soil. Specifically, the leafy vegetable soil (cabbage and kale soils) exhibited higher levels of OM, AN, TN, TK, SU, PHO, bacterial alpha diversity, and a more complex and stable network compared to the melon and fruit vegetable soil (cucumber and tomato soils). Based on these findings, we conclude that the cultivation of leafy vegetables is more advantageous for establishing a healthy soil microbial community compared to melon and fruit vegetables. These results provide valuable guidance for the management of vegetable cultivation in karst areas, promoting sustainable agricultural development.

## Electronic supplementary material

Below is the link to the electronic supplementary material.

Supplementary Material 1

Supplementary Material 2

## Data Availability

The raw reads of sequencing data is available at NCBI BioProject SRA database. under the accession number PRJNA865810.

## List of abbreviations

OM: organic matter
AN: alkaline hydrolyzable nitrogen
TN: total nitrogen
AP: available phosphorus
TP: total phosphorus
AK: available kalium
TK: total kalium
UR: urease
CA: catalase
SU: sucrase
