# Supplementary material for: Types of vegetables shape composition, diversity, and co-occurrence networks of soil bacteria and fungi in karst areas of southwest China
Source: BMC Microbiol. 2023 Jul 19;23:194. doi: 10.1186/s12866-023-02929-3 (PMC10354930; doi:10.1186/s12866-023-02929-3)
Supplement: Supplementary file 2 — Supplementary Material 2 [file 12866_2023_2929_MOESM2_ESM.doc]

**
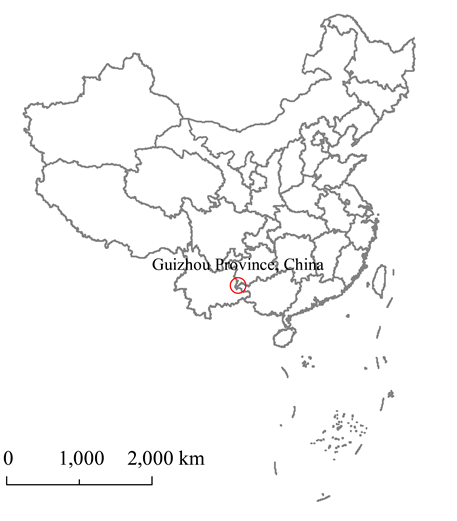
**

**Figure S1** Satellite imagery of sampling point. Notes: this Map was visualized with ArcGIS software.

**

**

**Figure S2** Keystone taxa of co-occurrence network in the leafy vegetable soil and melon and fruit vegetable soil of karst area. A represents keystone taxa of bacterial-fungal network in the leafy vegetable soil; B represents keystone taxa of bacterial-fungal network in melon and fruit vegetable soil; C represents keystone taxa of bacterial-bacterial network in the leafy vegetable soil; D represents keystone taxa of fungal-fungal network in the leafy vegetable soil; E represents keystone taxa of bacterial-bacterial network in the melon and fruit vegetable soil; F represents keystone taxa of fungal-fungal network in the melon and fruit vegetable soil.
